# The Construction and Comprehensive Analysis of ceRNA Networks and Tumor-Infiltrating Immune Cells in Bone Metastatic Melanoma

**DOI:** 10.3389/fgene.2019.00828

**Published:** 2019-09-25

**Authors:** Runzhi Huang, Zhiwei Zeng, Guangyu Li, Dianwen Song, Penghui Yan, Huabin Yin, Peng Hu, Xiaolong Zhu, Ruizhi Chang, Xu Zhang, Jie Zhang, Tong Meng, Zongqiang Huang

**Affiliations:** ^1^Department of Orthopaedics, The First Affiliated Hospital of Zhengzhou University, Zhengzhou, China; ^2^Division of Spine, Department of Orthopedics, Tongji Hospital affiliated to Tongji University School of Medicine, Shanghai, China; ^3^Tongji University School of Medicine, Tongji University, Shanghai, China; ^4^Department of Orthopedics, Shanghai General Hospital, School of Medicine, Shanghai Jiaotong University, Shanghai, China; ^5^Shanghai East Hospital, Key Laboratory of Arrhythmias, Ministry of Education, Tongji University School of Medicine, Shanghai, China

**Keywords:** melanoma, bone metastasis, competing endogenous RNA network, immune cell, nomogram

## Abstract

**Background/Aims:** As a malignant and melanocytic tumor, cutaneous melanoma is the devastating skin tumor with high rates of recurrence and metastasis. Bone is the common metastatic location, and bone metastasis may result in pathologic fracture, neurologic damage, and severe bone pain. Although metastatic melanoma was reported to get benefits from immunotherapy, molecular mechanisms and immune microenviroment underlying the melanoma bone metastasis and prognostic factors are still unknown.

**Methods:** Gene expression profiling of 112 samples, including 104 primary melanomas and 8 bone metastatic melanomas from The Cancer Genome Atlas database, was assayed to construct a ceRNA network associated with bone metastases. Besides, we detected the fraction of 22 immune cell types in melanoma *via* the algorithm of “cell type identification by estimating relative subsets of RNA transcripts (CIBERSORT).” Based on the significant ceRNAs or immune cells, we constructed nomograms to predict the prognosis of patients with melanoma. Ultimately, correlation analysis was implemented to discover the relationship between the significant ceRNA and immune cells to reveal the potential signaling pathways.

**Results:** We constructed a ceRNA network based on the interaction among 8 pairs of long noncoding RNA–microRNA and 15 pairs of microRNA–mRNA. CIBERSORT and ceRNA integration analysis discovered that AL118506.1 has both significant prognostic value (*P* = 0.002) and high correlation with T follicular helper cells (*P* = 0.033). Meanwhile, T cells CD8 and macrophages M2 were negatively correlated (*P* < 0.001). Moreover, we constructed two satisfactory nomograms (area under curve of 3-year survival: 0.899; 5-year survival: 0.885; and concordance index: 0.780) with significant ceRNAs or immune cells, to predict the prognosis of patients.

**Conclusions:** In this study, we suggest that bone metastasis in melanoma might be related to AL118506.1 and its role in regulating thrombospondin 2 and T follicular helper cells. Two nomograms were constructed to predict the prognosis of patients with melanoma and demonstrated their value in improving the personalized management.

## Introduction

Cutaneous melanoma is a malignant, melanocytic tumor and considered as the most harmful skin cancer (Cymerman et al., 2016; Lombard et al., 2019). All over the world, it accounts for about 232,100 (1.7%) cases of all newly diagnosed primary malignant cancers (excluding nonmelanoma), and meanwhile approximately 55,500 (0.7%) deaths are derived from cutaneous melanoma each year (Schadendorf et al., 2018). Nowadays, its incidence rate is still escalating dramatically (Schadendorf et al., 2019).

Extensive local resection with clean margins, depending on Breslow thickness of the tumor tissue, is recommended as the primary treatment for localized disease [The Cancer Genome Atlas (TCGA), 2015]. However, distant metastases often occur even after complete tumor resection due to the aggressive nature. Bone is the common metastatic location, and bone metastasis often results in pathologic fracture, neurologic damage, and severe bone pain, which decreases the quality of life (Braeuer et al., 2014; Bier et al., 2016). Regarding some patients with metastasis, systemic therapies such as targeted therapy and immunotherapy have achieved promising survival outcome; however, prognosis remains poor in most patients with metastasis (Bostel et al., 2016). Hence, it is in a desperate need to explore the molecular mechanism and probe for the prognostic factors for cutaneous melanoma patients with bone metastasis. The relationship among microRNA (miRNA), long noncoding RNA (lncRNA), and mRNA, known as ceRNA networks, had been explored in many diseases. However, ceRNA network mechanism underlying melanoma and bone metastasis still remains unknown.

In this study, we constructed a ceRNA network based on the gene expression profiling retrieved from the TCGA database to identify the ceRNAs associated with melanoma and bone metastasis. Besides, we perform “The Cell Type Identification by Estimating Relative Subsets of RNA Transcripts algorithm (CIBERSORT)” algorithm to detect the immune cells and their proportions in tumor tissues of melanoma. Additionally, nomograms were developed to predict the prognosis of melanoma with bone metastasis based on significant immune cells and ceRNA. The relationship between bone metastasis–related immune cells and ceRNA networks was evaluated to identify the underlying signaling pathways.

## Materials and Methods

### Data Collection and Differential Gene Expression Analysis

The Ethics Committee of the First Affiliated Hospital of Zhengzhou University approved this study (no. 2019-KY-107). We downloaded the RNA profiles of the primary melanomas and bone metastasis samples from the TCGA (https://tcga-data.nci.nih.gov/tcga/) database. HTseq-count and fragments per kilobase of exon per million reads mapped profiles of 112 samples, including 104 primary melanomas and 8 melanomas with bone metastasis, were assembled. Meanwhile, demographic and survival information of each patient was collected. The edgeR method was used to find differentially expressed mRNAs, lncRNAs, and miRNAs after removing nonmelanoma-specific expression genes (no expression in both the experimental group and control group). Only when the false discovery rate (FDR) *P* < 0.05 and the log (fold change) > 1.0 or <−1.0 could be regarded as differentially expressed gene of downregulation and upregulation, respectively.

### The Construction of the ceRNA Network

Prior to the initial statistical analysis, the miRNA–mRNA and lncRNA–miRNA interaction data were retrieved from miRTarBase (http://mirtarbase.mbc.nctu.edu.tw/) (Chou et al., 2018) and Incbase v.2 Experimental Module (http://carolina.imis.athena-innovation.gr/diana_tools/web/index.php?r=lncbasev2%2Findex-experimental) (Paraskevopoulou et al., 2016), respectively. Afterward, miRNAs, which illustrate significant outcomes in the aspect of regulating both IncRNAs and mRNAs in hypergeometric testing and correlation analysis, were collected for establishing the ceRNA network by Cytoscape v.3.5.1 (Shannon et al., 2003).

### Survival Analysis and Nomograms of Key Members in the ceRNA Network

Kaplan–Meier (K-M) survival analysis was performed to show the relationship between the expression level of biomarkers with the prognostic value illustrated in the ceRNA network and survival outcomes in patients with melanoma. Afterward, the significant biomarkers were incorporated into the reduced Cox proportional hazards model by screening the significant variables in the initial Cox models to illustrate the variables with prognostic values. Besides, Lasso regression (least absolute shrinkage and selection operator regression), which is a kind of linear regression using shrinkage where data values are shrunk to a specific point, was implemented to confirm the fitness of the established multifactor models. Ultimately, a nomogram based on the multivariable models was developed to predict the prognosis of patients with melanoma. In accordance with the expression level of biomarkers with prognostic values, we can acquire the points of each biomarker and add up to obtain the total points, which can display the 3- and 5-year overall survival probability. Meanwhile, calibration curves and receiver operating characteristic (ROC) curves were performed to evaluate the discrimination and precision of the nomogram.

### CIBERSORT Estimation

CIBERSORT is an analytical tool constructed by Newman et al. (2015) to identify the richness and proportion of the diversified cell types in a mixed cell population using gene expression data. Every cell type and their quantity in each sample can be conveniently acquired *via* CIBERSORT estimation. In this study, we use CIBERSORT algorithm to further probe for the cytological causes of molecular mechanisms of the pivotal biomarkers in the ceRNA network. The proportions of 22 immune cell types in the primary melanoma and melanoma with bone metastasis were estimated by CIBERSORT. Only when the CIBERSORT output of *P* < 0.05 could put the samples into further analysis. The Wilcoxon rank-sum test was performed to look for the significant immune cells in the aspect of the fraction between the primary melanoma and melanoma with bone metastasis. Then, K-M survival analysis was used to demonstrate the relationship between the overall survival of melanoma patients and proportion of specific immune cells. After being well filtered by Lasso regression, specific immune cells were incorporated into the Cox proportional hazards model. Then, nomogram was constructed to predict the prognosis for melanoma. Concordance index of Cox model was applied to access the discrimination and accuracy of the nomogram. Ultimately, Pearson correlation analysis was carried out to show the relationship between immune cells and biomarkers.

### Online Database Validation

To minimize bias caused by the imbalanced sample size and get more complete annotation of key biomarkers, multiple online databases including the CellMarker (Zhang et al., 2019), LncRNA2Target (Cheng et al., 2019), Ontogene (Cheng et al., 2016), String (Szklarczyk et al., 2019), DincRNA (Cheng et al., 2018), SurvExpress (Aguirre-Gamboa et al., 2013), Cancer Cell Line Encyclopedia (CCLE) (Ghandi et al., 2019), Genotype–Tissue Expression (GTEx) (Consortium, 2015), Oncomine (Elfilali et al., 2006), and Gene Expression Omnibus (GEO) (ID: GSE19234 (Bogunovic et al., 2009), GSE22153 (Jonsson et al., 2010) were used to detect gene expression levels of key biomarkers at the tissue and cellular levels.

### Statistical Analysis

Only two-sided *P* < 0.05 was defined as statistical significance. All the statistical analyses were performed with R version 3.5.1 software (Institute for Statistics and Mathematics, Vienna, Austria; www.r-project.org) (package: GDCRNATools (Li et al., 2018), edgeR, ggplot2, rms, glmnet, preprocessCore, survminer, timeROC).

## Results

### Identification of Significantly Differentially Expressed Genes

**Figure 1:**
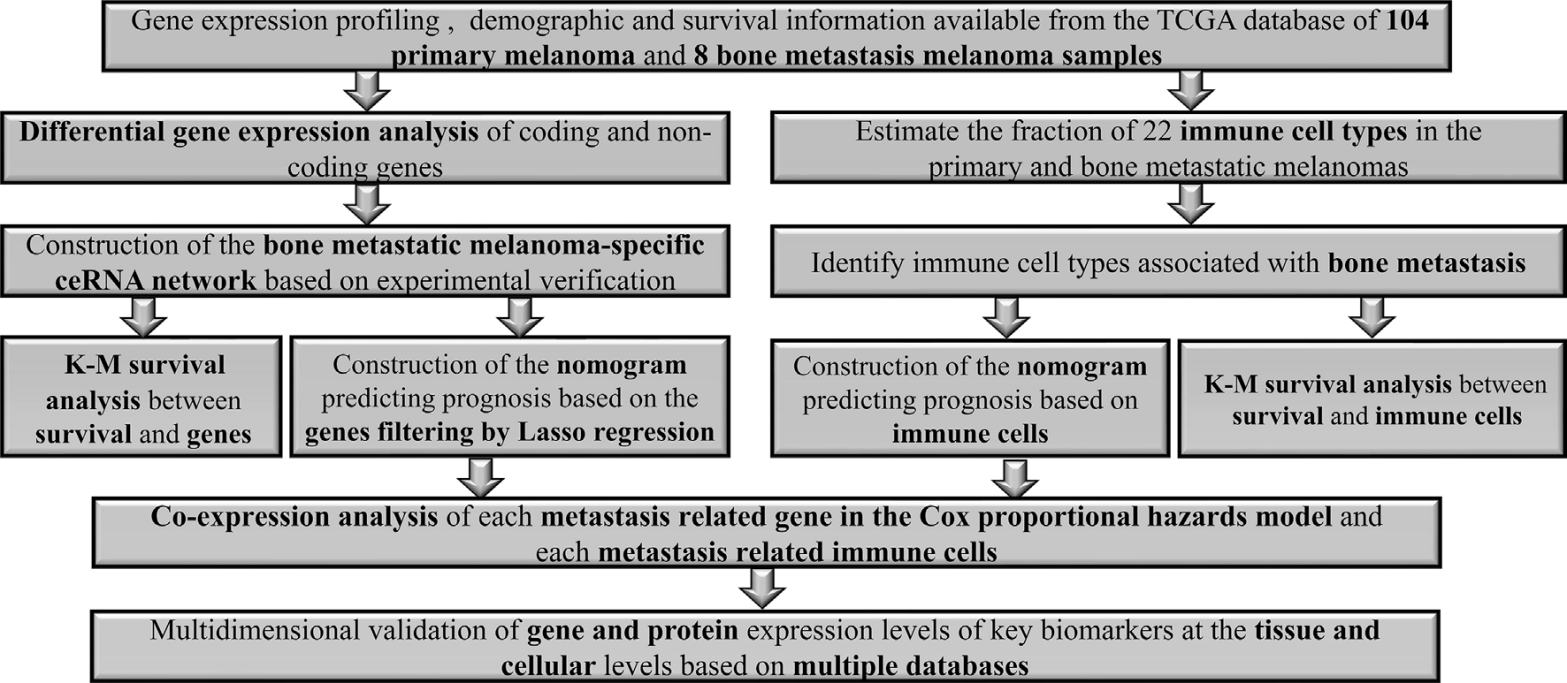
The flowchart of the analysis process.

**Figure 2:**
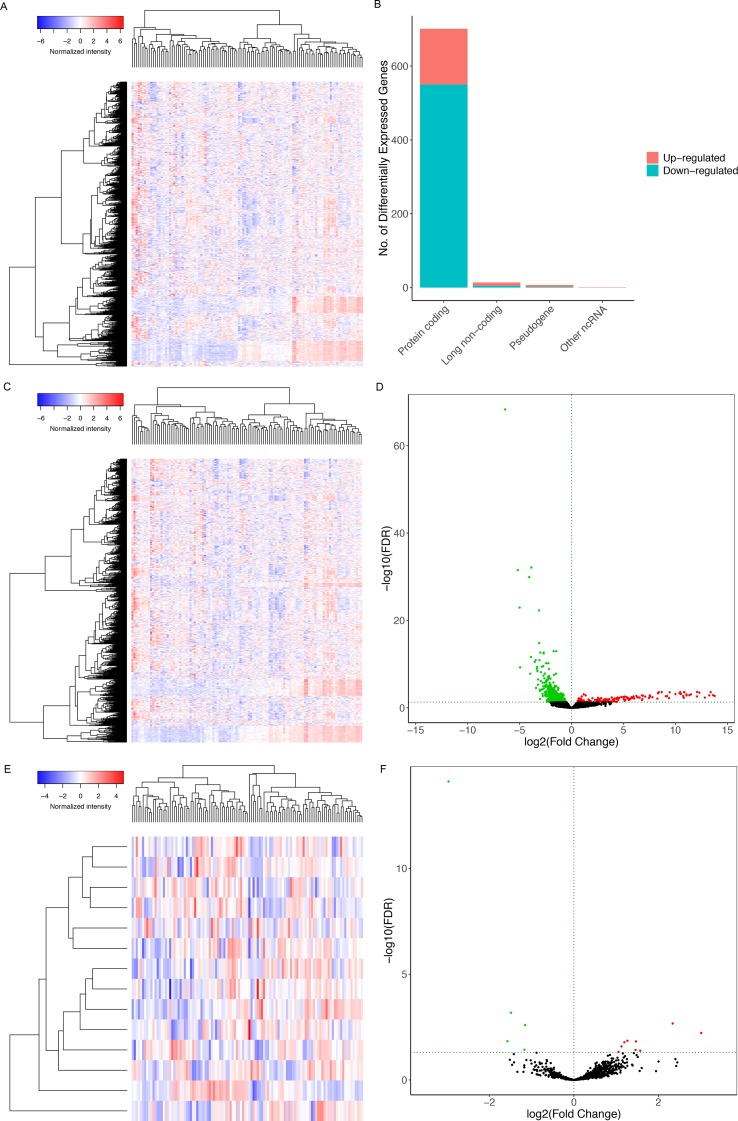
The heat maps of differentially expressed **(A)** RNAs, **(C)** miRNAs, **(E)** lncRNAs between the bone-metastatic melanoma and the primary melanoma. **(B)** Bar plot showing differentially expressed protein-coding genes, long noncoding genes, pseudogenes, and other RNAs. Red and blue represent up-regulated and down-regulated RNAs, respectively. It shows that 550 of 701 differentially expressed protein-coding genes are down-regulated and 151 are up-regulated. Besides, among 14 differentially expressed lncRNAs, 5 lncRNAs are down-regulated, and 9 are up-regulated. Volcano plots of differentially expressed mRNAs **(D)** and lncRNAs **(F)**. We defined the log (fold change) >1.0 or <−1.0 and FDR <0.05 as the critical point. Thus, the red and blue dots in the plots represent high and low expression RNAs with statistical significance, respectively. Meanwhile, black dots represent mRNAs and lncRNAs without statistical significance between the primary and the bone-metastatic melanoma.

**Figure 1** illustrates the analysis process of this study. The baseline features of all the patients retrieved from the TCGA database were described in **Table S1**. We defined the log (fold change) >1.0 or < −1.0 and FDR <0.05 as the critical point and found out that there were 701 differentially (550 down- and 151 up-) expressed protein-coding genes, along with 14 differentially (5 down- and 9 up-) expressed lncRNAs and 72 differentially (45 down- and 27 up-) expressed miRNAs between the bone metastatic melanoma and the primary melanoma from the TCGA database (**Figures 2A–F**).

### ceRNA Network Establishment and Survival Analysis

**Figure 3:**
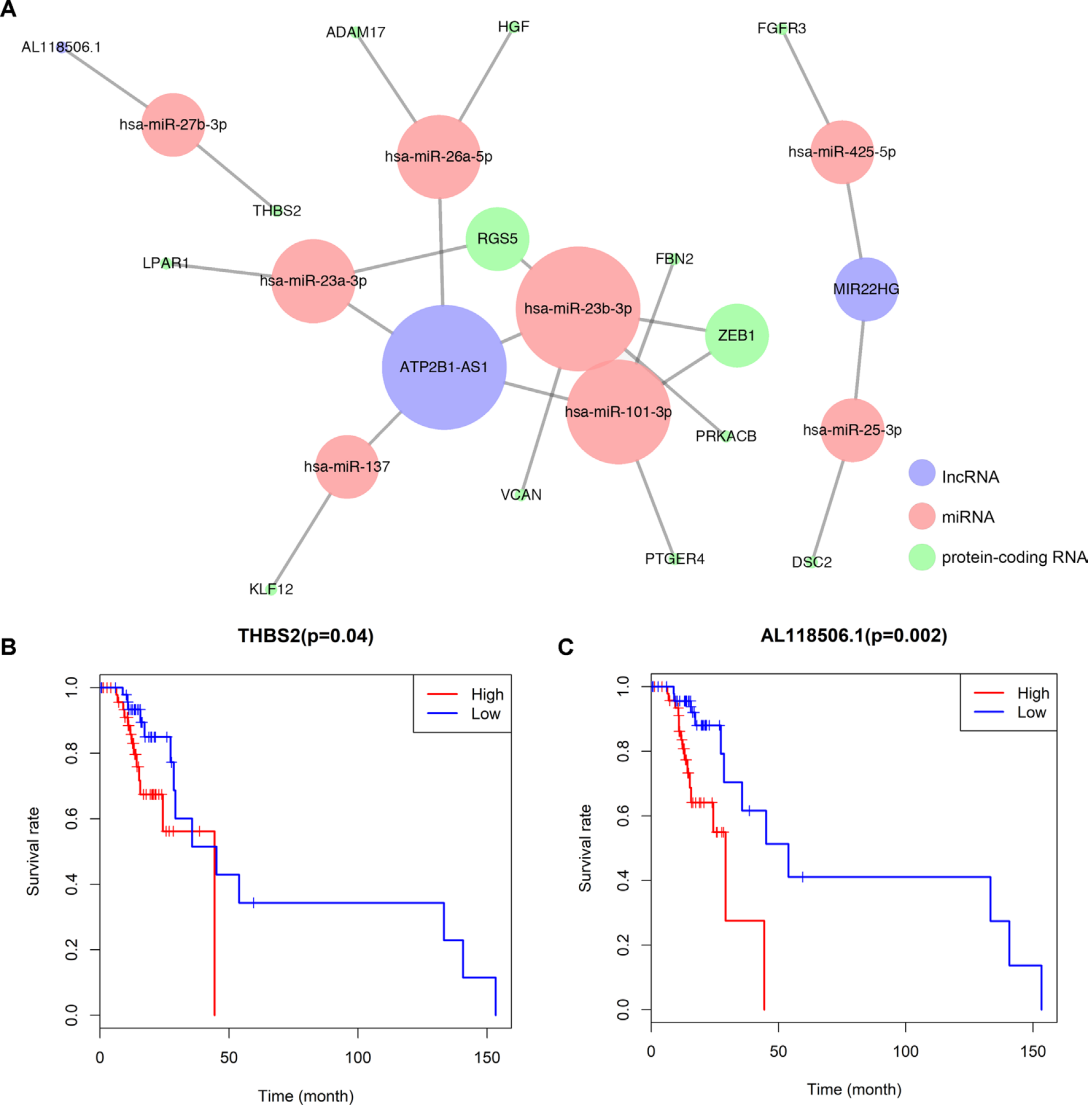
**(A)** Overview of the lncRNA–miRNA–mRNA ceRNA network of melanoma with 8pairs of lncRNA–miRNA and 15 pairs of miRNA–mRNA. Red balls represent miRNAs, blue balls represent lncRNAs, and green balls represent protein-coding RNAs. Kaplan–Meier survival curves based on the expression of biomarkers involved in ceRNA network related to the bone metastasis in melanoma shows that **(B)** THBS2 (*P* = 0.040) and **(C)** AL118506.1 (*P* = 0.002) had significantly prognostic values.

A ceRNA network was established based on the interaction among 8 pairs of lncRNA–miRNA and 15 pairs of miRNA–mRNA (**Figure 3A**) (**Table 1**). Kaplan–Meier survival analysis was implemented to explore the relationship between the prognosis and biomarkers involved in ceRNA network related to the bone metastasis in melanoma. The results revealed that thrombospondin 2 (THBS2) (*P* = 0.040) and AL118506.1 (*P* = 0.002) displayed significance (**Figures 3B, C**). According to enrichment analysis, the significant genes associated with bone metastasis in melanoma were mostly functioned in extracellular matrix organization (**Figure S1**).

**Table 1:** Hypergeometric testing and correlation analysis results of ceRNAs network.

LncRNA	Protein-coding RNA	MiRNAs	Correlation *P*	Hypergeometric test *P*
AL118506.1	THBS2	hsa-miR-27b-3p	0.006581855	0.00747894
MIR22HG	FGFR3	hsa-miR-425-5p	0.022787186	0.006234399
MIR22HG	DSC2	hsa-miR-25-3p	0.000396455	0.001248439
ATP2B1-AS1	RGS5	hsa-miR-23a-3p,hsa-miR-23b-3p	2.58E−06	0.001872829
ATP2B1-AS1	FBN2	hsa-miR-101-3p	0.000158704	0.006866417
ATP2B1-AS1	KLF12	hsa-miR-137	0.009365181	0.020470827
ATP2B1-AS1	VCAN	hsa-miR-23b-3p	0.001403378	0.006866417
ATP2B1-AS1	LPAR1	hsa-miR-23a-3p	5.78E−09	0.020470827
ATP2B1-AS1	ZEB1	hsa-miR-101-3p,hsa-miR-23b-3p	2.43E−05	0.014703227
ATP2B1-AS1	HGF	hsa-miR-26a-5p	0.000176262	0.033905608
ATP2B1-AS1	PTGER4	hsa-miR-101-3p	0.016481894	0.006866417
ATP2B1-AS1	PRKACB	hsa-miR-23b-3p	9.06E−07	0.006866417
ATP2B1-AS1	ADAM17	hsa-miR-26a-5p	0.000901194	0.033905608

ceRNAs, competing endogenous RNAs; LncRNA, long noncoding RNA; MiRNA, microRNA.

### Construction of the Prediction Model Based on the ceRNA Network

**Figure 4:**
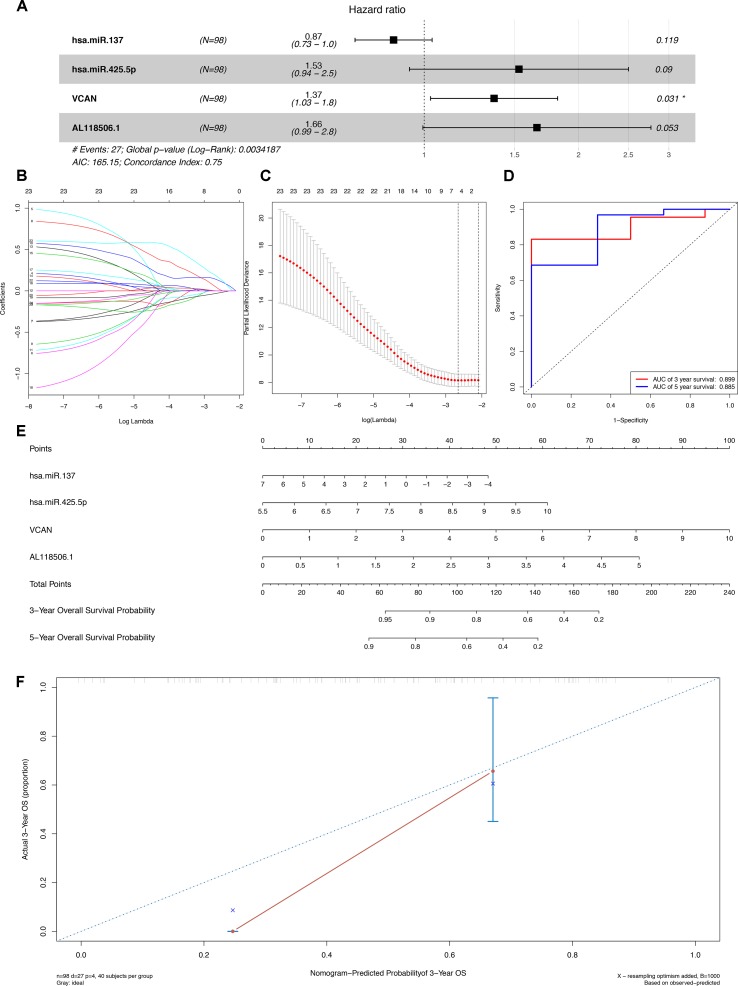
**(A)** The Cox proportional hazards model based on RNAs selected by **(B) (C)** Lasso regression. hsa-miR-137, hsa-miR-425-5p, VCAN, and AL118506.1 are incorporated into the Cox proportional hazards model. **(E)** Nomogram for predicting patients’ outcome based on RNAs (hsa-miR-137, hsa-miR-425-5p, VCAN, and AL118506.1) in Panel **(A)**. **(D)** ROC curves and **(F)** calibration curves for assessing the discrimination and accuracy of the nomogram. Besides, AUCs of the 3- and 5-year survival were 0.899 and 0.855, respectively. AUC, area under curve; ROC, receiver operating characteristic.

The outcomes of Lasso regression illustrated that four genes, hsa-miR-137, hsa-miR-425-5p, VCAN, and AL118506.1, were critical to modeling and were then incorporated into the Cox regression, after which the nomogram, aimed to predict the prognosis, was constructed according to the Lasso regression. The areas under curve (AUC) of the 3- and 5-year survival were 0.899 and 0.855, respectively, which reflects the satisfactory accuracy. Additionally, the discrimination of the nomogram was suggested by the calibration curves (**Figures 4A–F**).

### Immune Cells Related to the Melanoma

**Figure 5:**
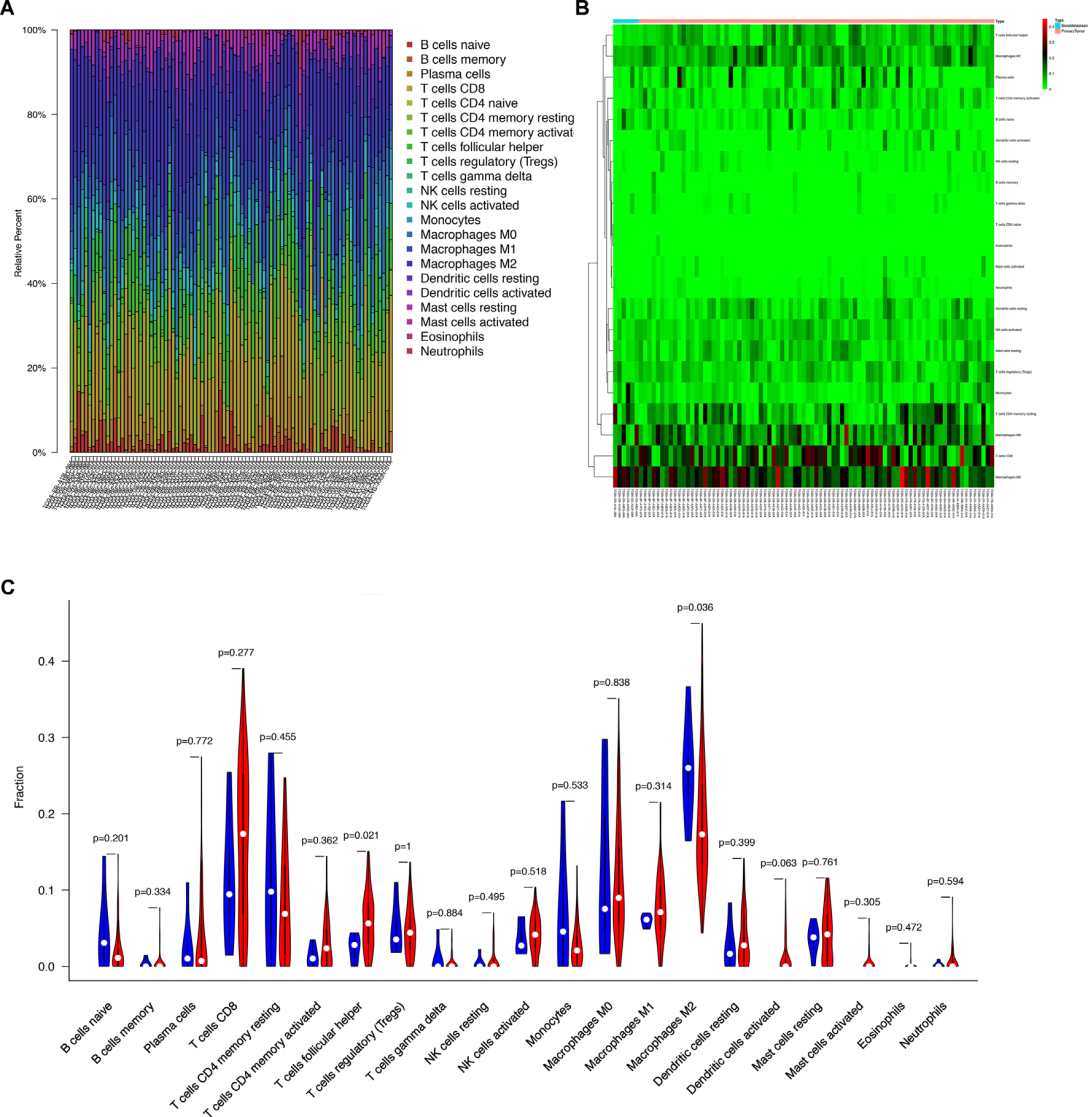
**(A)** Bar plot showing cell types and relative percent in melanoma tissues. Different colors represent different cell types, which are listed in the right as *y* axis, while *x* axis represents different samples. **(B)** Heat map of tumor-infiltrating cells in tumor tissues in patients with the primary melanoma and the bone metastatic disease. Annotations on top show clustering of samples. While the blue represents the melanoma with bone metastasis, the red symbolizes the primary melanoma. **(C)** Violin plot for comparing cells’ proportion between the primary and bone-metastatic disease. It illustrates that the proportion of the T follicular helper (Tfh) cells in the melanoma with bone metastasis was relatively less than that in the primary melanoma (*P* = 0.021), and macrophages M2 was relatively greater in the melanoma with bone metastasis (*P* = 0.036).

The composition of the immune cells in the melanoma evaluated by CIBERSORT algorithm was illustrated in the histogram and the heat map (**Figures 5A, B**). The results of the Wilcoxon rank-sum test revealed that the proportion of the T follicular helper (Tfh) cells in the melanoma with bone metastasis was relatively less than that in the primary melanoma (*P* = 0.021), and macrophages M2 was relatively greater in the melanoma with bone metastasis (*P* = 0.036) (**Figure 5C**).

### Construction of the Prediction Model Based on the Immune Cells

**Figure 6:**
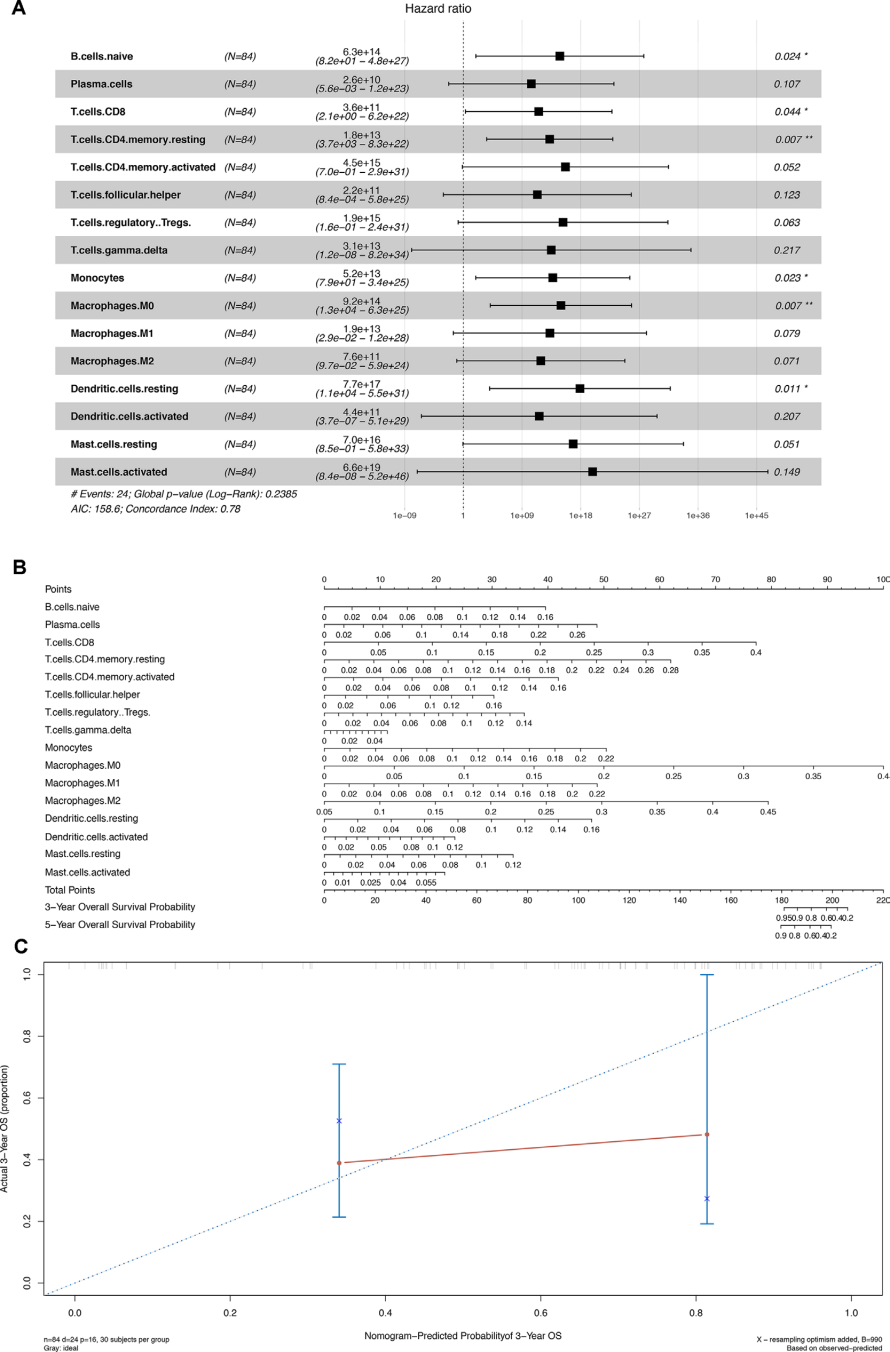
**(A)** Cox proportional hazards model integrated by 16 different types of immune cells. **(B)** Nomogram for predicting patients’ outcome based on 16 cells in Panel **(A)**. **(C)** Calibration curves for evaluating the accuracy of the nomogram. *P < 0.05; **P < 0.001.

Similarly, 16 of 22 immune cells, which showed significant prognostic values in the initial Cox regression model, were integrated into the final multivariable model with satisfactory predictive power (concordance index 0.780) and were utilized to construct the nomogram (**Figures 6A, B**). The concordance curve and concordance index showed a good concordance of the model (**Figure 6C**). Based on the result of the Kolmogorov–Smirnov test, the fraction of regulatory T cells (Tregs) in stages T1, T2, T3, and T4 showed significant difference between patients with or without bone metastasis (**Figure S2**).

### Comprehensive Analysis of Genes and Immune Cells

**Figure 7:**
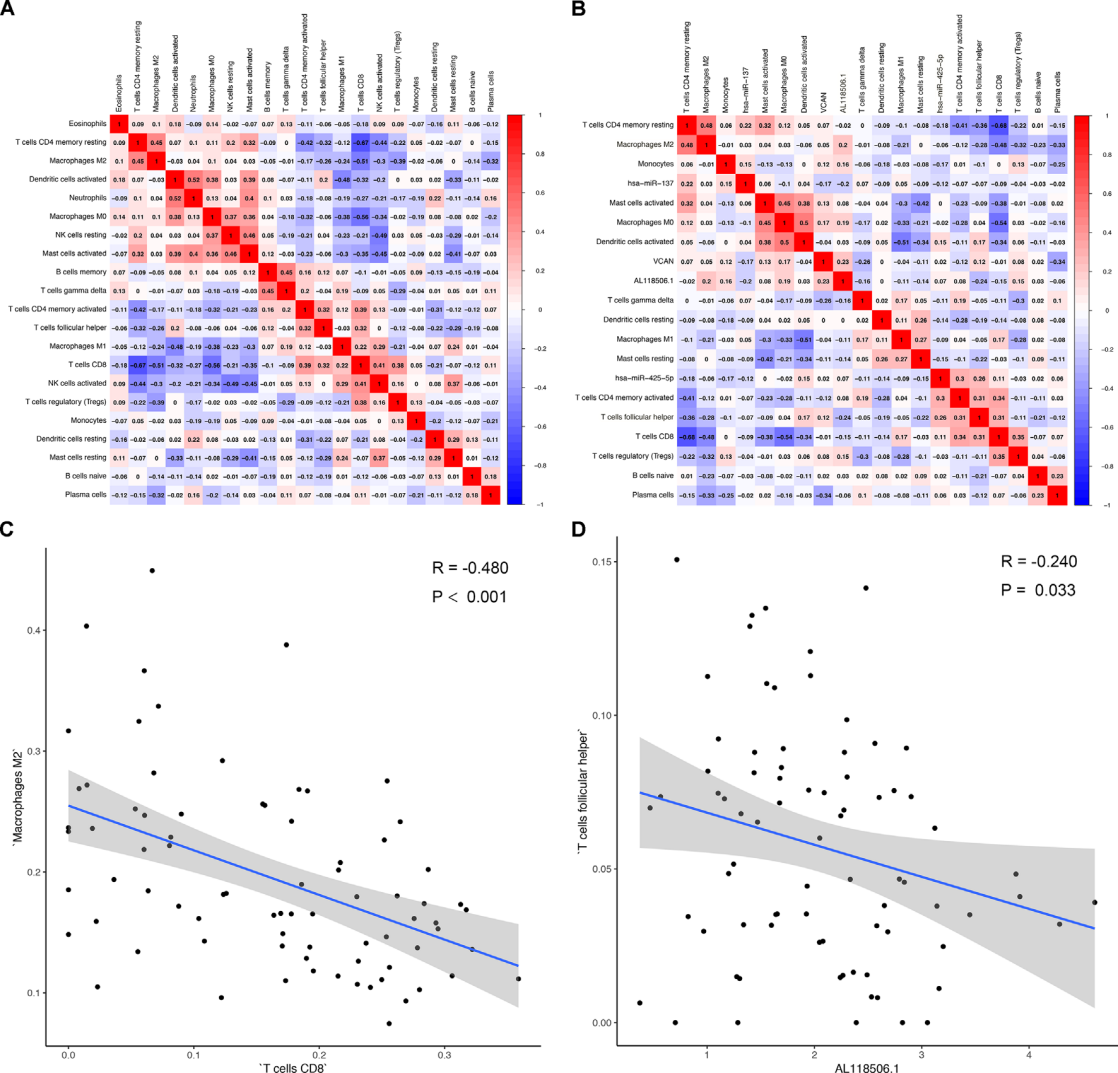
**(A)** Correlation analysis (Pearson analysis) of different tumor-infiltrating cells and **(B)** the relationships between different tumor-infiltrating cells and differentially expressed genes in tumor tissues of melanoma. Scatterplots further illustrate the exact relationship between T cells CD8 and macrophages M2 (*P* < 0.001, *R* = −0.480) **(C)**, AL118506.1, and T follicular helper cells (*P* = 0.033, *R* = −0.240) **(D)**. Besides, gray-shaded areas in two graphs represent the standard errors of the blue regression lines. R, correlation coefficient.

Correlation analysis (Pearson analysis) was applied to demonstrate the coexpression patterns among diversified immune cells (**Figure 7A**). Likewise, correlation relationship (Pearson analysis) between immune cells and biomarkers was further analyzed and illustrated (**Figure 7B**). As shown, hsa-miR-425-5p and Tfh cells (*P* = 0.019, *R* = 0.260) (**Figure 7C**), AL118506.1 and Tfh cells (*P* = 0.033, *R* = −0.240) (**Figure 7D**), and Tfh cells and hsa-miR-425-5p (**Figure S3**) represented good correlation. Eventually, bone metastasis–specific immune cells and ceRNAs significantly associated with prognosis were integrated into one multivariable model and one nomogram (**Figure S4**), which could decently predict the prognosis of SKCM (AUC of 3-year survival: 1.000; AUC of 5-year survival: 1.000). However, the model diagnostic information suggested that the prediction model had bias due to the small sample size.

### Metastasis-Specific ceRNAs and Immune Cells’ Surface Markers Coding Genes Showing Significant Results in Multidimensional Validation

In order to explore the expressions of metastasis-specific ceRNAs and immune cells’ surface markers coding genes in different datasets, a dimensional validation applying multiple online databases was performed.

At the cellular level, BCL6 transcription repressor (BCL6), membrane metalloendopeptidase (MME), C-X-C motif chemokine ligand 13 (CXCL13), inducible T-cell costimulator (ICOS), and programmed cell death 1 (PDCD1) had been reported as the surface markers of Tfh cell in the CellMarker (**Figure S5**). AL118506.1 is a type of lncRNA (Ensemble ID: ENSG00000268858). According to DincRNA, Ontogene, and LncRNA2Target database, AL118506.1 is the antisense to Abhydrolase domain containing 16B (ABHD16B, also known as C20orf135), and it can down-regulate the expression level of hsa-miR-27b-3p. However, the function of AL118506.1 remains largely unknown. Thus, AL118506.1, ABHD16B, THBS2, BCL6, MME, CXCL13, ICOS, and PDCD1 were incorporated into further multidimensional validation.

First, **Figure S6** illustrates the protein–protein interaction network of these genes, indicating that there are many interactions between THBS2 protein and T infertile helper cell’s surface markers. Besides, in the CCLE and GTEx, we found that THBS2 was expressed in various SKCM cell lines, and Tfh cell’s surface marker coding gene expressions were low, while in normal skin tissue THBS2 and AL118506.1 were expressed, and surface marker coding gene expressions were also low (**Figures S7A, S7C**). Meanwhile, significant coexpression relationships between THBS2 and Tfh cell’s surface marker coding genes had been observed in tissue levels, but not in cancer cell lines (**Figures S7B, S7D**). Besides, in meta-analysis of Oncomine, THBS2 (Median rank 1,088, *P* < 0.001) (**Figures S8A, B**), ICOS (Median rank 1,008, COPA = 1.854) (**Figures S8C, D**), CXCL13 (Median rank 536.5, COPA = 30.145) (**Figures S8E, F**), BCL6 (Median rank 434.5, COPA = 2.016) (**Figures S8G, H**), MME (median rank 221.0, COPA = 8.940) (**Figures S8I, J**), and PDCD1 (median rank 7,680, *P* = 0.350) (**Figures S8C, D**) all showed significant results in multiple melanoma–related studies except PDCD1. Additionally, the reanalysis results of GSE19234 (**Figure S9**) and GSE22153 (**Figure S10**) in SurvExpress suggested that these genes have significant predictive value for metastasis (censoring event: metastasis, hazard ratio = 5.19 [95% confidence interval {CI}, 1.92–14.05], *P* = 0.001, **Figure S9C**) (censoring event: subcutaneous metastasis, hazard ratio = 4.01 [95% CI, 1.93–8.34], *P* < 0.001, **Figures S10C, D**) and prognosis (censoring event: overall death, hazard ratio = 3.15 [95% CI, 1.71–5.80], *P* < 0.001, **Figure S10B**).

## Discussion

Malignant melanoma is regarded as one of the most devastating and metastatic diseases with a drastic increasing incidence rate around the world (Bostel et al., 2016). Tumor metastasis is the advanced stage of disease and its complications often decrease the quality of life, especially for the bone metastasis. Although the mechanisms of tumorigenesis and metastasis are still unclear for melanoma, molecular and cellular features often changed during the process and are often viewed as important predictors (Braeuer et al., 2014; Rodina et al., 2016). Thus, the differentially expressed genes and tumor-infiltrating immune cells in the primary melanoma and bone metastasis attract our interest, which is seldom focused by previous studies.

In the current study, we first figured out the differently expressed and statistically significant ceRNA and tumor-infiltrating immune cells between the primary and metastatic melanoma. Afterward, two nomograms are constructed based on them to predict the outcomes of patients with melanoma. The high AUC value and concordance index in two nomograms might contribute to make an evaluation for bone metastasis and survival outcomes. At last, according to the results of K-M survival analysis and correlation analysis, we inferred that the ceRNA regulatory mechanism of AL18506.1 (lncRNA), THBS2 (mRNA), hsa-miR-27b-3p (miRNA), and Tfh cell might play a crucial role in bone metastasis of melanoma.

Recently, a myriad of studies had uncovered that no more than 2% of the whole genome encode protein-coding genes, which suggests that most of the human transcriptomes are represented by noncoding RNAs (Volders et al., 2013). mRNAs, miRNAs, and lncRNAs are connected through the competitive endogenous RNA networks in an intricate crosstalk (Tay et al., 2014). The interaction among miRNA, lncRNA, and mRNA, operating as ceRNA networks, had been drastically explored in many diseases, including lung cancer, gastric cancer, and gallbladder cancer, among others (Kumar et al., 2014; Chen et al., 2018; Chen et al., 2019). However, ceRNA network mechanism underlying melanoma and bone metastasis remains largely unknown. In our study, we identified that AL118506.1 (lncRNA) could down-regulate and up-regulate the level of hsa-miR-27b-3p and THBS2, respectively, to promote bone metastasis in patients with melanoma *via* ceRNA network. The role of hsa-miR-27b-3p was shown to be essential in malignant transformation, which is in conformity with our present study (Liu et al., 2015).

Thrombospondins (THBSs) had been verified to play important roles in various processes, including angiogenesis, cellular adhesion, extracellular matrix interaction, tumor formation, and metastasis (Roberts, 2008; Liu et al., 2018). Thrombospondin 2, one of members in THBSs, is revealed to regulate the antiangiogenic activity and prevent the development of focal adhesion in endothelial cells (Agostini et al., 2012). Moreover, the overexpression of THBS2 had been demonstrated to be positively correlated with node metastasis and over survival in many types of cancer, including colorectal adenocarcinoma, myxoid liposarcoma, prostate cancer, and gastric cancer (Kim et al., 2010; Slavin et al., 2014; Chang et al., 2016; Lin et al., 2016; Nezu et al., 2016; Zhuo et al., 2016; Qian et al., 2017; Wei et al., 2017). The role of THBS2 was also investigated in melanoma in a previous study, and metastatic uveal melanoma had a higher expression level of THBS2, which is consistent with our analysis (Liu and Ma, 2018).

We also found out the different proportions of numerous immune cells in the primary melanoma and bone metastatic melanoma tissues. T follicular helper cells and macrophages M2 were demonstrated to be related to bone metastasis. The nomogram, composed of 16 kinds of immune cells, was constructed to predict the overall survival, which showed the great clinical utility with the concordance index of 0.78.

Generally, the CD8+ cytotoxic T cell is considered to be the main element of active antitumor immunity, whose full function greatly relied on adequate help from CD4+ T cells (Gillgrass et al., 2014). Naive CD4+ T cells could differentiate into different T helper (T_H_) cells, including T_H_1, T_H_2, T_H_17, Tregs, and Tfh cells (Zhu et al., 2010). The Tfh cell is one subtype of CD4+ T cells, which is defined by its surface phenotypes with the highest expression level of CXCR5(Vinuesa et al., 2016). It had been demonstrated that Tfh plays an important part in the construction of humoral immunity through regulating the formation and cellular reactions that happen in the germinal center (Qi, 2016). The dysregulated Tfh cells were found to be associated with several autoimmune or (and) immune-deficient diseases, including systemic lupus erythematosus, HIV, and lymphoma (Tangye et al., 2013). A few previous studies had revealed that there are ordered lymph node–like structures mainly formed by Tfh cells in extensively infiltrated tumors, including breast cancer, lung cancer, and colorectal cancer, with obviously detectable Tfh cells, which function in antitumor immunity with positive clinical outcome (Dieu-Nosjean et al., 2008; deLeeuw et al., 2012). Other human-related studies also identified that Tfh cells had great capacity in directly assisting B cells through releasing interleukin 21 (IL-21), and IL-21 could further help human antigen-specific cytotoxic T cells to generate and proliferate, which also suggests that Tfh cells had a direct antitumorigenic function (Chen et al., 2016). Thus, patients with fewer Tfh cells had a decreased immune response in fighting against tumor, while immunosuppression was positively correlated with tumor metastasis (Bidwell et al., 2012). In our study, our data indicate that Tfh cells had a lower expression level in patients with bone metastatic disease.

Similarly, the importance of CD4+ cells of high concentration in hindering melanoma metastasis and recurrence has also been reported (He et al., 2017). Antibody of anti–programmed death 1, situated on the surface of CD4+ cells, had been verified to prove the clinical outcomes of patients with melanoma (Yamaguchi et al., 2018). Additionally, the expression levels of tumor-infiltrating cells of CD8 and macrophages M2 are, to some extent, related to clinical outcomes. The extensively studied immune infiltrate in different cancer had established that macrophages M2 could suppress antitumor immunity and promote tumor progression (Gillgrass et al., 2014; Guerriero et al., 2017). The data presented in this study also showed that macrophages M2 expression is higher in samples of patients with bone metastasis. Furthermore, the correlation analysis led us to know that the level of macrophages M2 was inversely correlated with that of CD8 T cells, and patients with more CD8 cells in tumor tissues had worse outcome, which was highly consistent with a previous study (Gillgrass et al., 2014).

The correlation analysis revealed that Tfh cells were associated with AL118506.1 (*R* = −0.240, *P* = 0.033). Based on the results of correlation analysis and hypergeometric testing of ceRNA network, AL118506.1 (lncRNA), THBS2 (protein-coding RNA), and hsa-miR-27b-3p (miRNA) were considerably correlated (*P* = 0.007). Therefore, we inferred that the interaction among hsa-miR-27b-3p, AL118506.1, THBS2, and Tfh cells was highly relevant with bone metastasis in patients with melanoma.

Nevertheless, there are several unavoidable limitations to our study that should be taken into consideration. First, the quantity of related data available from the public datasets is still limited. The idea of acquiring the same number of cases in the aspects of different genders, age groups, and races, among others, to decrease the potential error and bias is far too difficult to be achieved under the current circumstances, which leads to the lack of comprehensiveness of this study. Second, we have not taken into account the heterogeneity of the immune microenvironment associated with the location of immune infiltration. Third, all data series retrieved for the construction of nomograms aimed to predict outcomes were from the west. Therefore, if patients are from other countries, samples are tested by other platforms, but GPL96 or GPL570. Last but not least, the small sample size of bone metastasis melanoma may reduce the confidence and transformation of the predictive models into other cohorts. And to minimize bias, additional validation based on multiple databases was applied to detect gene expression levels of key biomarkers at the tissue and cellular levels, showing the key biomarkers were significantly associated with metastasis and prognosis of SKCM (**Figure S5**–**S10**).

## Conclusions

According to ceRNA networks and tumor-infiltrating immune cells, two nomograms were built, respectively, in our study to predict survival and metastasis of melanoma patients and had great utility, which was verified by high concordance index and AUC values. Based on the comprehensive clinical information from the prediction nomograms, individual management of melanoma patients could be greatly improved. Furthermore, with sufficient evidence shown in this study, we speculate that melanoma bone metastasis may depend on the interaction among hsa-miR-27b-3p, AL118506.1, THBS2, and Tfh cells.

## Data Availability

All datasets for this study are included in the TCGA-SKCM program.

## Ethics Statement

The Ethics Committee of the First Affiliated Hospital of Zhengzhou University approved this study (no. 2019-KY-107).

## Author Contributions

Conception/design: RH, ZZ, GL, DS, PY, HY, PH, XiZ, RC, XuZ, TM, JZ, and ZH. Provision of study material: RH, ZZ, GL, TMeng, JZ, and ZH. Collection and/or assembly of data: RH, ZZ, GL, DS, PY, HY, PH, XiZ, RC, and XuZ. Data analysis and interpretation: RH, ZZ, GL, DS, PY, HY, PH, XiZ, RC, and XuZ. Manuscript writing: RH, ZZ, GL, TM, JZ, and ZH. Final approval of manuscript: RH, ZZ, GL, DS, PY, HY, PH, XiZ, RC, XuZ, TM, JZ, and ZH.

## Funding

This study was supported in part by the National Natural Science Foundation of China (grant no. 81702659; 81772856; 81501203). Youth Fund of Shanghai Municipal Health Planning Commission (No.2017YQ054); Henan Medical Science and Technology Research Project (grant no. 201602031).

## Conflict of Interest Statement

The authors declare that the research was conducted in the absence of any commercial or financial relationships that could be construed as a potential conflict of interest.

## Abbreviations

AUC, Area under curve; ceRNA, competitive endogenous RNA; lncRNA, long noncoding RNA; miRNA, microRNA; CIBERSORT, Cell type identification by estimating relative subsets of RNA transcripts; TCGA, The Cancer Genome Atlas; FDR, false discovery rate; SD, standard deviation; ROC, Receiver operating characteristic curves; THBS, Thrombospondin, Tfh, T follicular helper cells; IL-21, interleukin 21.

## Supplementary Material

Table S1 Baseline information of 112 patients diagnosed with Primary melanoma.

Figure S1 The result of enrichment analysis showed that genes in melanoma tissues were significantly associated with extracellular matrix organization.

Figure S2 The results of the Wilcoxon rank-sum test in T regulatory cells (Tregs) of different T stages.

Figure S3 The correlation analysis revealed that T cells follicular helper was positively correlated with hsa-miR-425-5p (*P* = 0.019; *R* = 0.260).

Figure S4 The results of Cox proportional hazards model and the nomogram integrating both biomarkers and immune cell portions significantly associated with prognosis. Bone metastasis–specific immune cells and ceRNAs significantly associated with prognosis were integrated into one multi-variable model and one nomogram **(A, E)**, which could decently predict the prognosis of SKCM (AUC of 3-year survival: 1.000; AUC of 5-year survival: 1.000) **(D)**. However, the model diagnostic information suggested that the prediction model had bias due to the small sample size **(A, B, C, F)**.

Figure S5 Use CellMarker to explore the surface markers of T follicular helper cells. At the cellular level, BCL6 transcription repressor (BCL6), membrane metalloendopeptidase (MME), C-X-C motif chemokine ligand 13 (CXCL13), inducible T-cell costimulator (ICOS) and Programmed cell death 1 (PDCD1) had been reported as the surface markers of T follicular helper cell in the CellMarker.

Figure S6 Protein–protein interaction network of ABHD16B, THBS2, BCL6, MME, CXCL13, ICOS, PDCD1, indicating that there are many interactions between THBS2 protein and T infertile helper cell’s surface markers.

Figure S7 The expression levels and co-expression analysis of AL118506.1, ABHD16B, THBS2, BCL6, MME, CXCL13, ICOS, PDCD1 in various SKCM cell lines and normal skin tissue in Cancer Cell Line Encyclopedia (CCLE) **(A, B)** and The Genotype–Tissue Expression (GTEx) database **(C, D)**.

Figure S8 Validation of THBS2 **(A, B)**, ICOS **(C, D)**, CXCL13 **(E, F)**, BCL6 **(G, H)**, MME **(I, J)**, and PDCD1 **(K, L)** on a transcriptional level in multiple cancer types and multiple studies using the Oncomine database.

Figure S9 The results of reanalysis of GSE19234 in SurvExpress. The reanalysis results of GSE19234 in SurvExpress suggested that these genes have significant predictive value for metastasis (Censoring event: metastasis, Hazard Ratio = 5.19 (95% CI, 1.92–14.05), *P* = 0.001)

Figure S10 The results of reanalysis of GSE22153 in SurvExpress. The reanalysis results of GSE22153 in SurvExpress suggested that these genes have significant predictive value for metastasis (Censoring event: subcutaneous metastasis, Hazard Ratio = 4.01 (95% CI, 1.93–8.34), *P*< 0.001) and prognosis (Censoring event: overall death, Hazard Ratio = 3.15 (95% CI, 1.71–5.80), *P*< 0.001).
