# Supplementary material for: The Construction and Comprehensive Analysis of ceRNA Networks and Tumor-Infiltrating Immune Cells in Bone Metastatic Melanoma
Source: Front Genet. 2019 Sep 25;10:828. doi: 10.3389/fgene.2019.00828 (PMC6774271; doi:10.3389/fgene.2019.00828)
Supplement: Table S1 — Baseline information of 112 patients diagnosed with Primary melanoma. [file Table_1.docx]

| Variables | Total Patients (N = 112) |
| --- | --- |
| **Age, years** |  |
| Mean ± SD | 63.69 ± 14.31 |
| Median (Range) | 63.5 (24 - 90) |
| **Gender** |  |
| Female | 70 (62.5%) |
| Male | 42 (37.5%) |
| **Race** |  |
| Asian | 7 (6.25%) |
| White | 103 (91.96%) |
| Unknown | 2 (1.79%) |
| **Bone metastasis** |  |
| Yes | 8 (7.14%) |
| No | 104 (92.86%) |

**Table S1** Baseline information of 112 patients diagnosed with Primary melanoma

**Abbreviations:** SD, Standard deviation.
