# Supplementary figures and images for: The Construction and Comprehensive Analysis of ceRNA Networks and Tumor-Infiltrating Immune Cells in Bone Metastatic Melanoma
Source: Front Genet. 2019 Sep 25;10:828. doi: 10.3389/fgene.2019.00828 (PMC6774271; doi:10.3389/fgene.2019.00828)

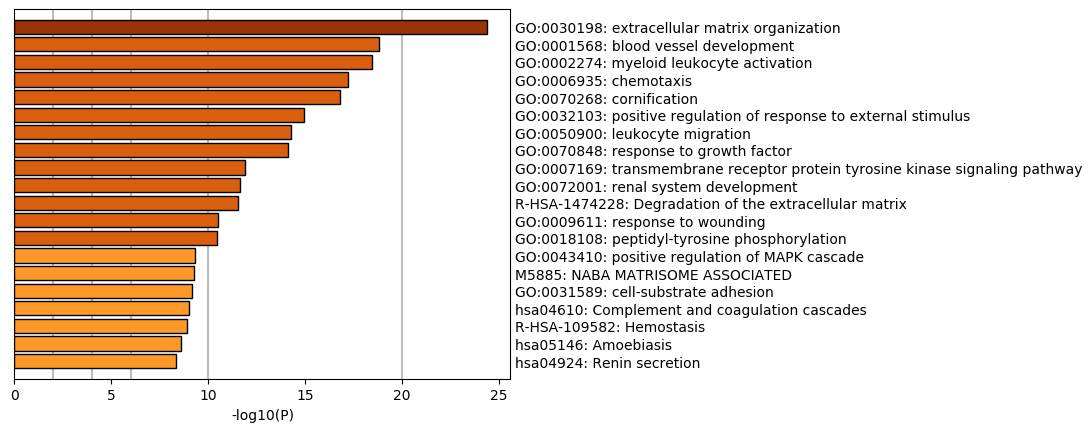

Supplement: Figure S1 — The result of enrichment analysis showed that genes in melanoma tissues were significantly associated with extracellular matrix organization. [file Image_1.tif]

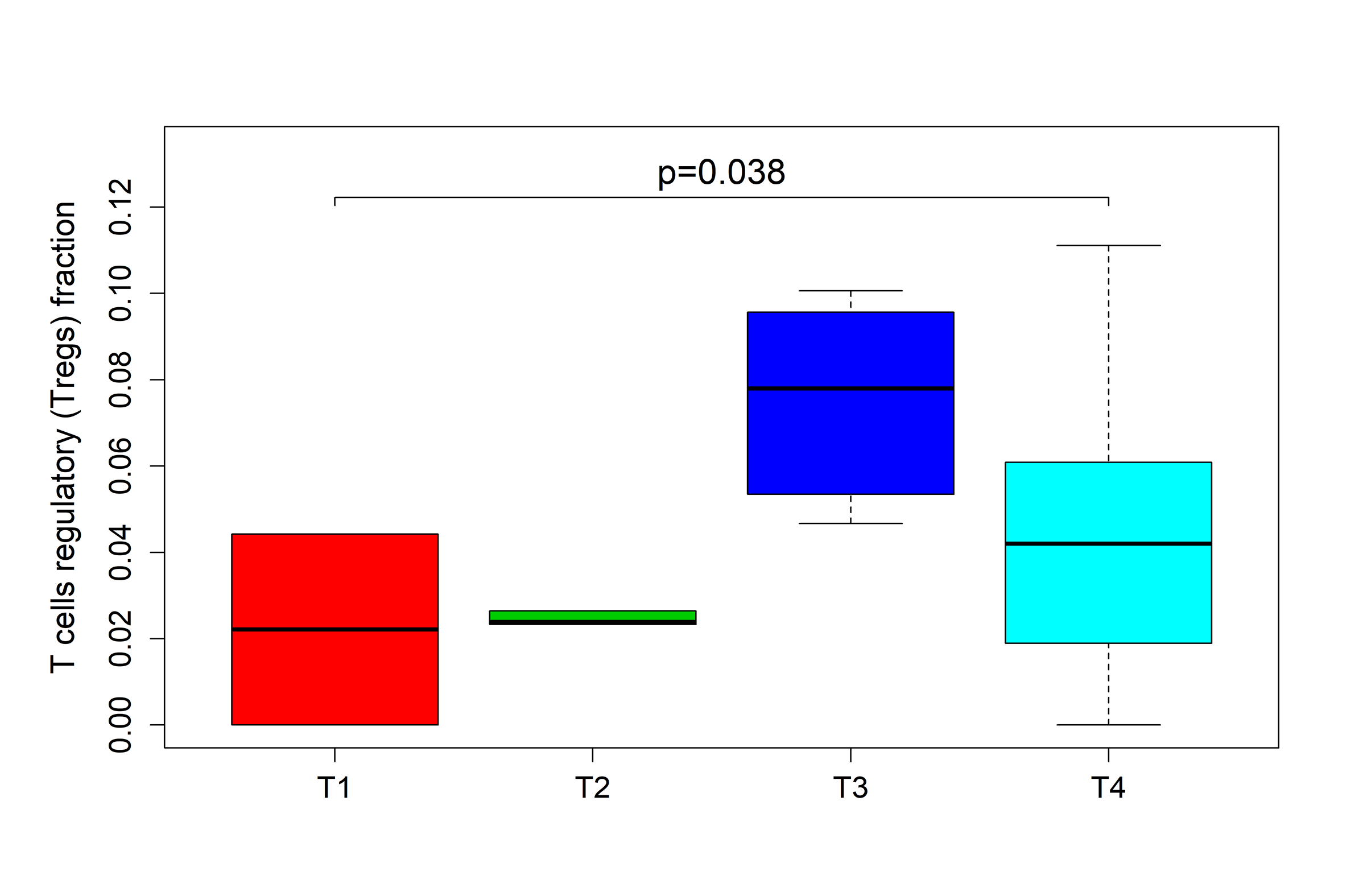

Supplement: Figure S2 — The results of the Wilcoxon rank-sum test in T regulatory cells (Tregs) of different T stages. [file Image_2.tif]

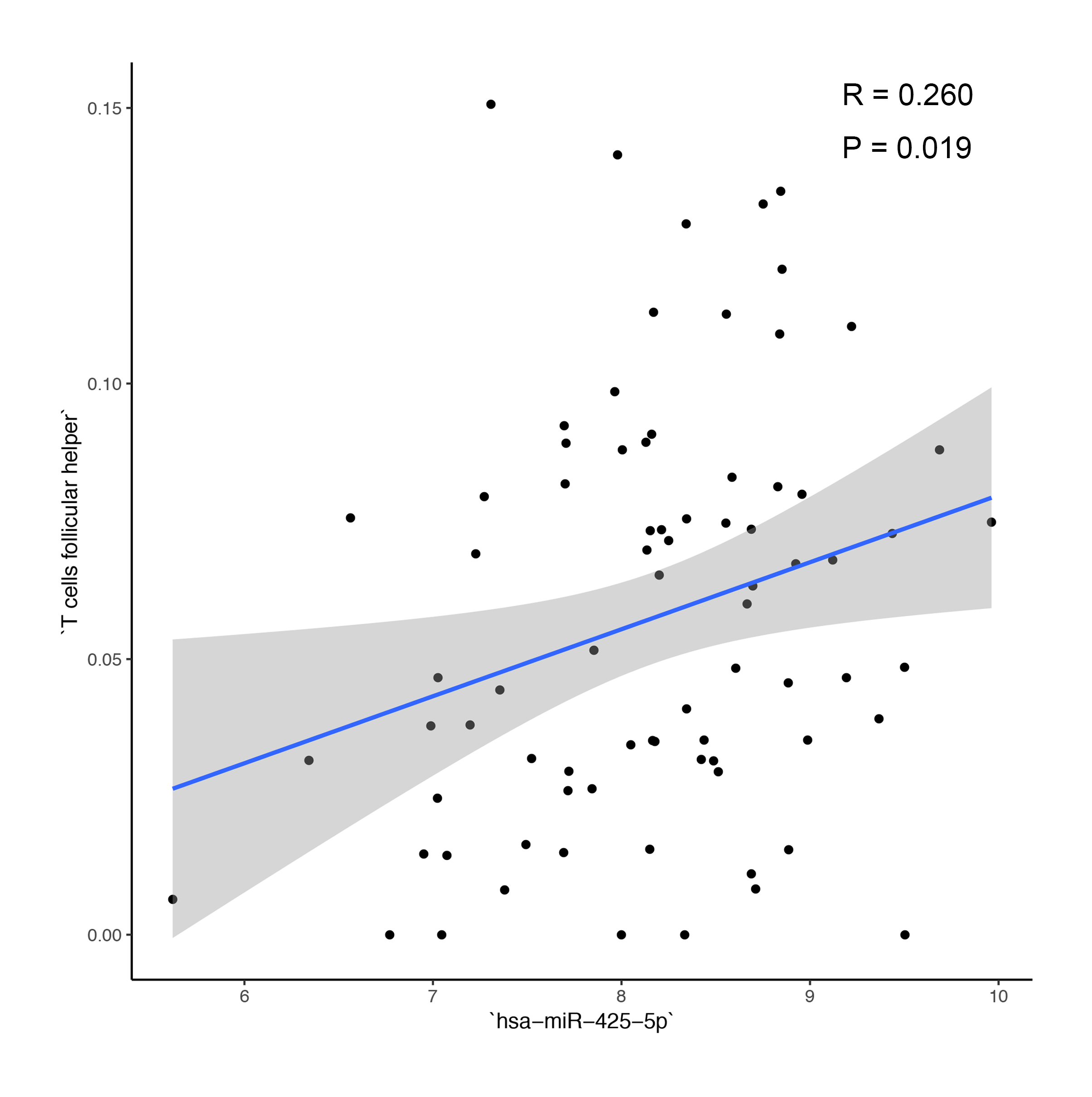

Supplement: Figure S3 — The correlation analysis revealed that T cells follicular helper was positively correlated with hsa-miR-425-5p (P = 0.019; R = 0.260). [file Image_3.tif]

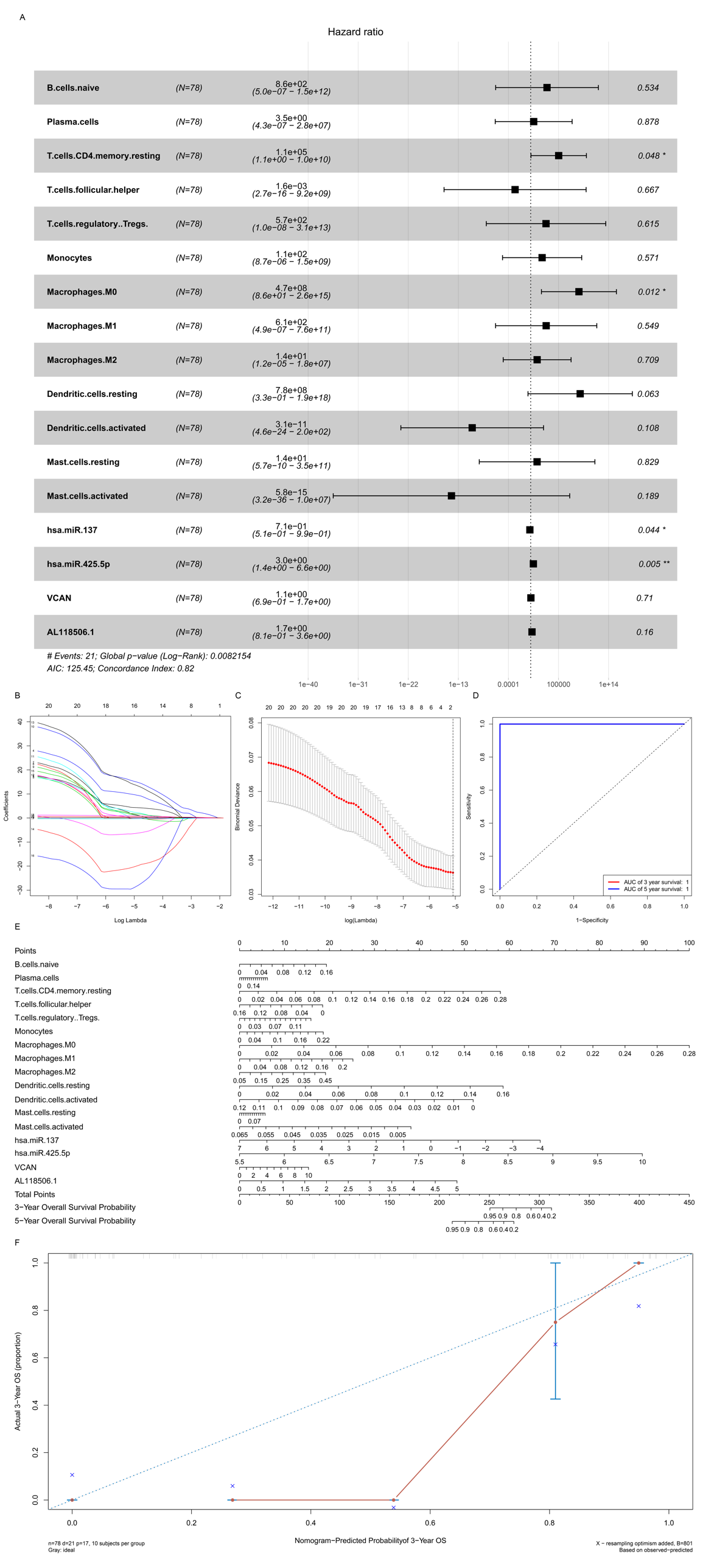

Supplement: Figure S4 — The results of Cox proportional hazards model and the nomogram integrating both biomarkers and immune cell portions significantly associated with prognosis. Bone metastasis–specific immune cells and ceRNAs significantly associated with prognosis were integrated into one multi-variable model and one nomogram (A, E), which could decently predict the prognosis of SKCM (AUC of 3-year survival: 1.000; AUC of 5-year survival: 1.000) (D). However, the model diagnostic information suggested that the prediction model had bias due to the small sample size (A, B, C, F). [file Image_4.tif]

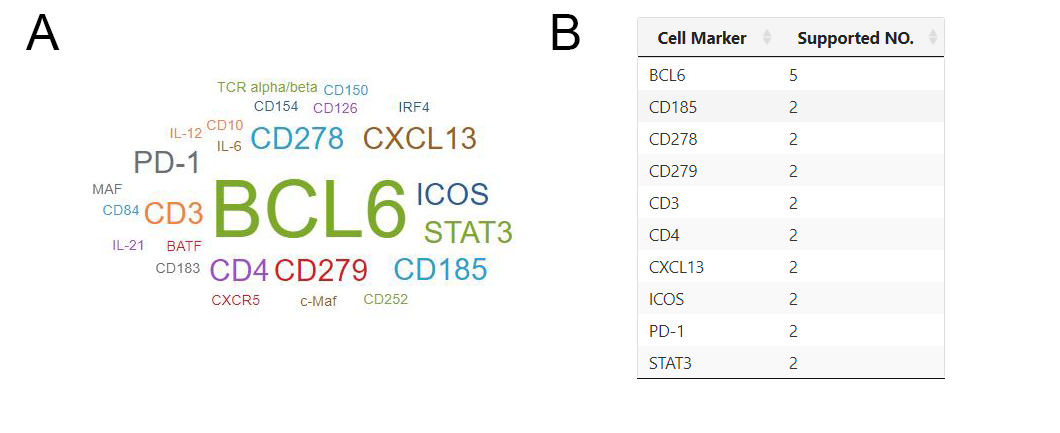

Supplement: Figure S5 — Use CellMarker to explore the surface markers of T follicular helper cells. At the cellular level, BCL6 transcription repressor (BCL6), membrane metalloendopeptidase (MME), C-X-C motif chemokine ligand 13 (CXCL13), inducible T-cell costimulator (ICOS) and Programmed cell death 1 (PDCD1) had been reported as the surface markers of T follicular helper cell in the CellMarker. [file Image_5.tif]

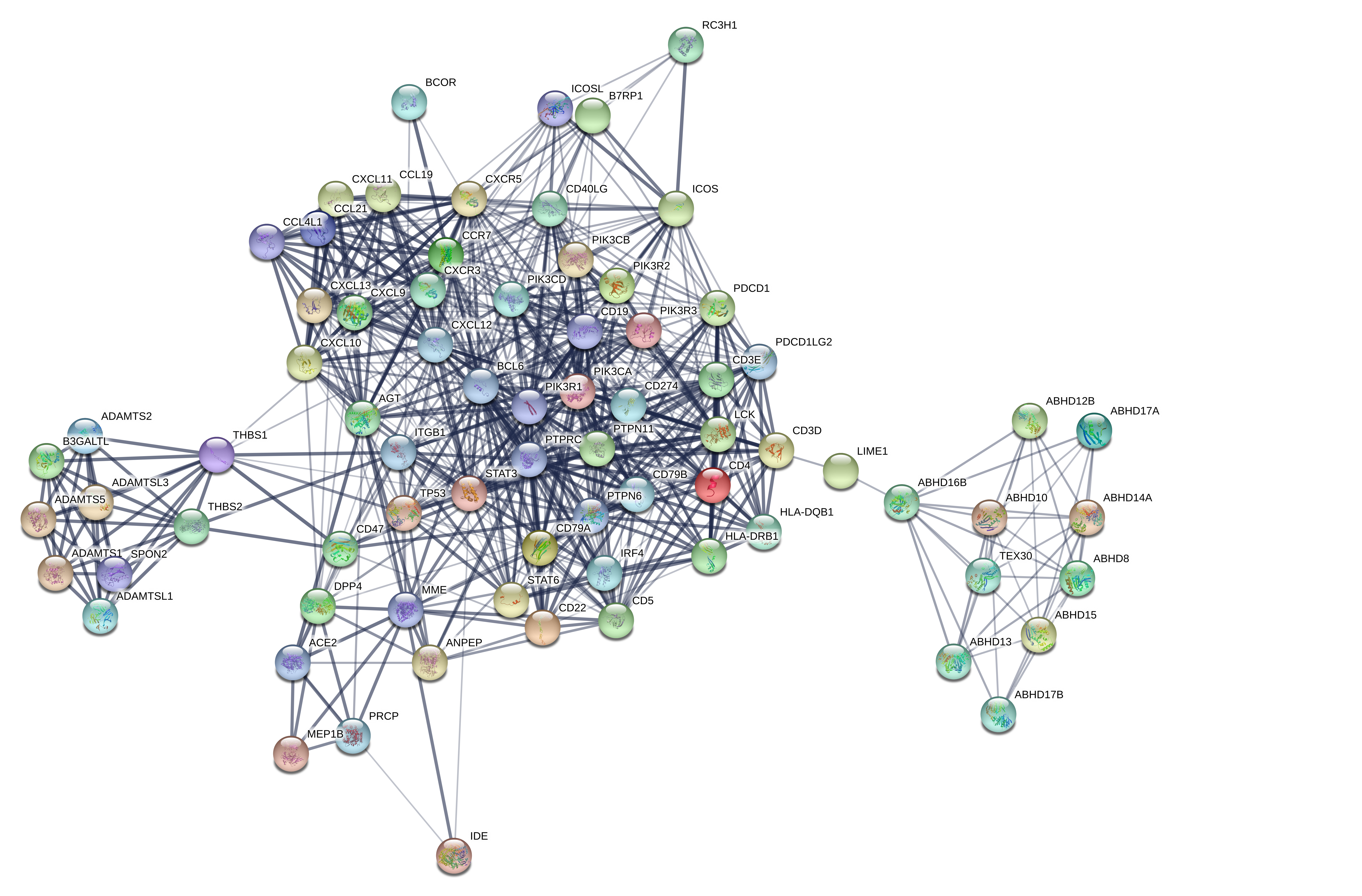

Supplement: Figure S6 — Protein–protein interaction network of ABHD16B, THBS2, BCL6, MME, CXCL13, ICOS, PDCD1, indicating that there are many interactions between THBS2 protein and T infertile helper cell’s surface markers. [file Image_6.tif]

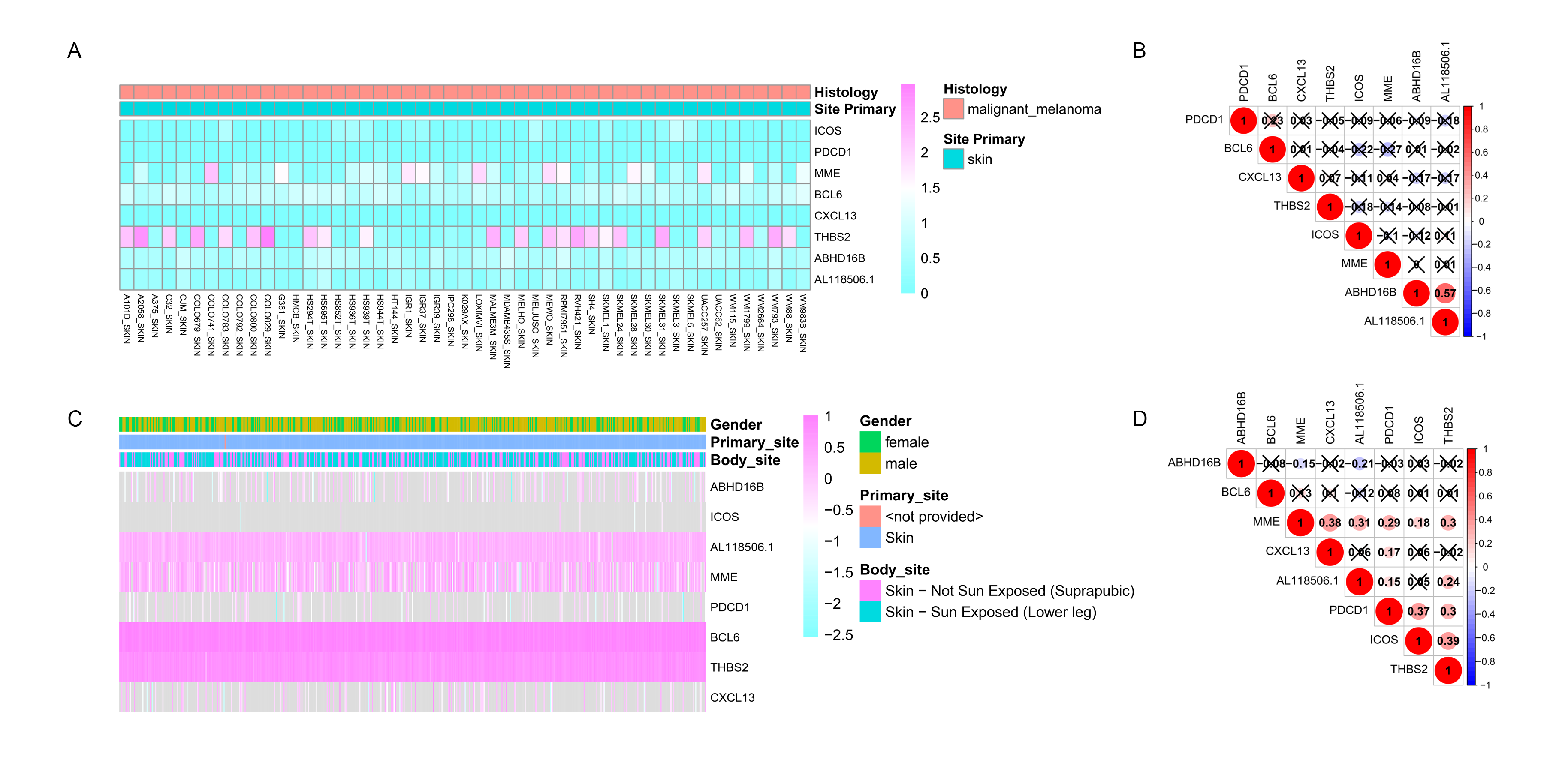

Supplement: Figure S7 — The expression levels and co-expression analysis of AL118506.1, ABHD16B, THBS2, BCL6, MME, CXCL13, ICOS, PDCD1 in various SKCM cell lines and normal skin tissue in Cancer Cell Line Encyclopedia (CCLE) (A, B) and The Genotype–Tissue Expression (GTEx) database (C, D). [file Image_7.tif]

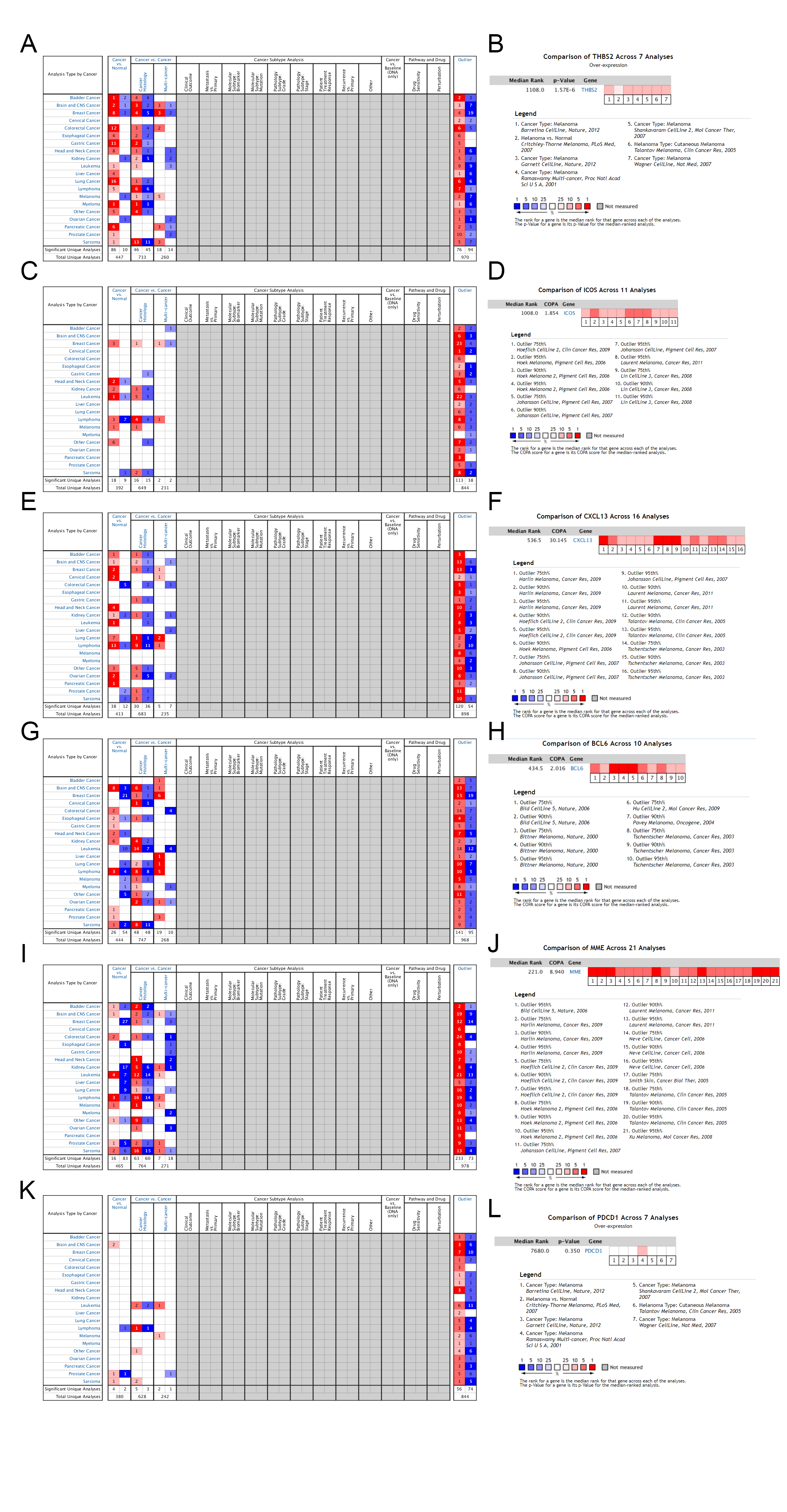

Supplement: Figure S8 — Validation of THBS2 (A, B), ICOS (C, D), CXCL13 (E, F), BCL6 (G, H), MME (I, J), and PDCD1 (K, L) on a transcriptional level in multiple cancer types and multiple studies using the Oncomine database. [file Image_8.tif]

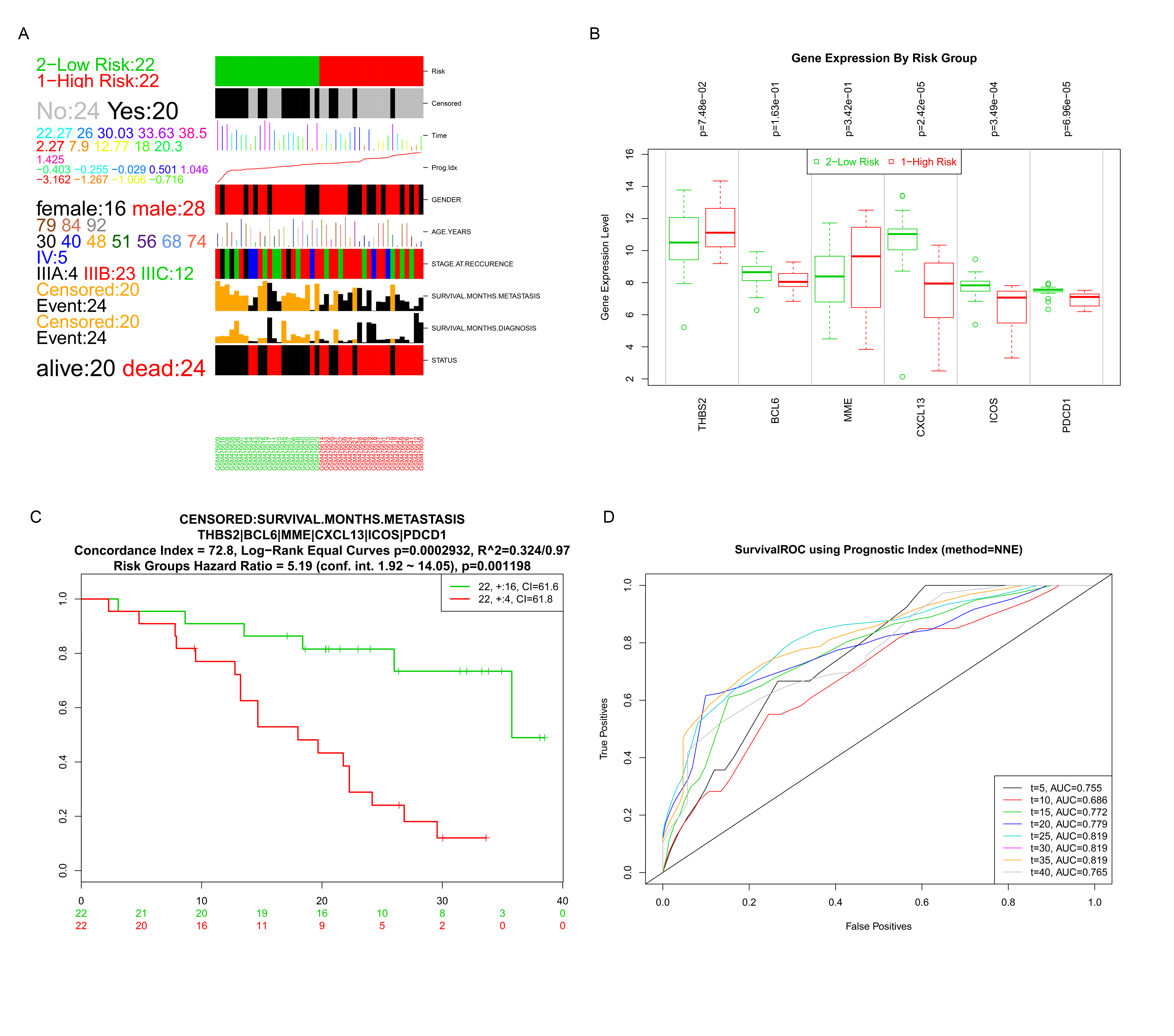

Supplement: Figure S9 — The results of reanalysis of GSE19234 in SurvExpress. The reanalysis results of GSE19234 in SurvExpress suggested that these genes have significant predictive value for metastasis (Censoring event: metastasis, Hazard Ratio = 5.19 (95% CI, 1.92–14.05), P = 0.001) [file Image_9.tif]

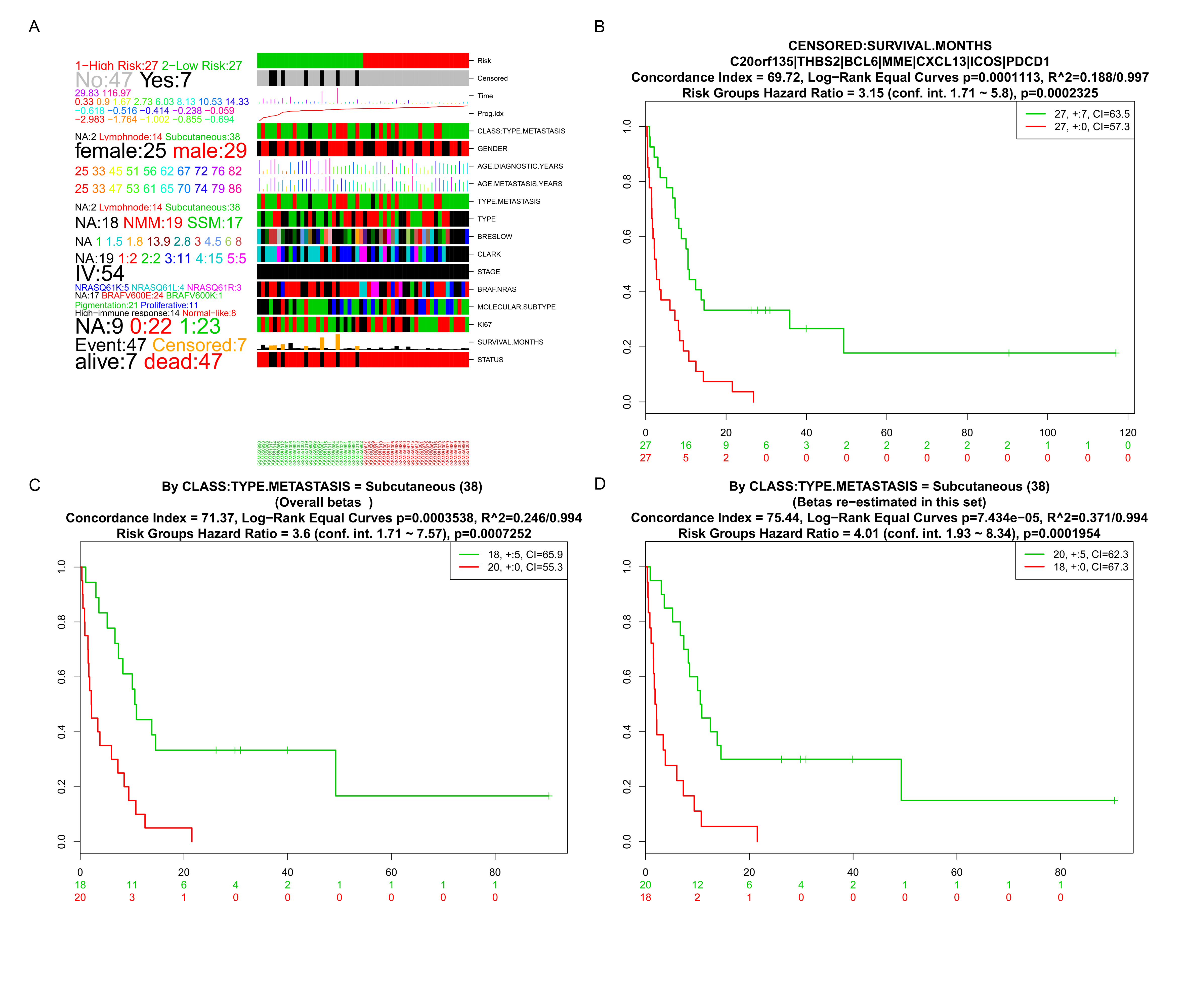

Supplement: Figure S10 — The results of reanalysis of GSE22153 in SurvExpress. The reanalysis results of GSE22153 in SurvExpress suggested that these genes have significant predictive value for metastasis (Censoring event: subcutaneous metastasis, Hazard Ratio = 4.01 (95% CI, 1.93–8.34), P< 0.001) and prognosis (Censoring event: overall death, Hazard Ratio = 3.15 (95% CI, 1.71–5.80), P< 0.001). [file Image_10.tif]
